# Prognostic value of des-γ-carboxy prothrombin in patients with hepatocellular carcinoma treated with transarterial chemotherapy: A systematic review and meta-analysis

**DOI:** 10.1371/journal.pone.0225170

**Published:** 2019-11-15

**Authors:** Ming Yang, Xuejun Zhang, Jinlong Liu

**Affiliations:** Department of Hepatobiliary Surgery, Affiliated Hospital of Chengde Medical University, Chengde, Hebei province, China; Texas A&M University, UNITED STATES

## Abstract

**Background:**

Serum des-γ-carboxy prothrombin (DCP) is a hepatocellular carcinoma (HCC) tumor marker that can be used to assess patient prognosis. Since the value of DCP in predicting the prognosis of hepatocellular carcinoma patients treated with transarterial chemotherapy remains controversial, we performed a meta-analysis of previous clinical studies.

**Methods:**

A systematic literature search was performed using PubMed, the MEDLINE database, EMBASE, and the Cochrane Library in accordance with the PRISMA guidelines. The hazard ratio (HR) with 95% confidence interval (CI) was used to estimate the effect size.

**Results:**

Six respective cohort studies including a total of 943 cases were identified. The pooled results showed that low DCP was associated with a favorable overall survival (OS)(HR 0.653, 95% CI 0.444–0.960), and DCP response was associated with increased OS (HR 0.387,95% CI 0.215–0.697) and progression-free survival (PFS) (HR 0.42,95% CI 0.23–0.74) in HCC patients treated with transarterial chemotherapy.

**Conclusions:**

DCP values in HCC patients undergoing hepatic arterial chemotherapy seem to be associated with OS and PFS. Thus, monitoring DCP values and observing the DCP response should be part of the management of patients undergoing transarterial chemotherapy.

## Introduction

Des-γ-carboxy-prothrombin (DCP) is an abnormal prothrombin in which one or more glutamic acid residues of the Gla domain are not fully carboxylated to gamma-carboxylated glutamate (Gla) in its molecular structure. As a result, it completely loses normal prothrombin function [1]. This substance is also referred to as a protein induced by vitamin K deletion or antagonist-II (PIVKA-II) due to the lack of vitamin K or antagonist-II protein. In 1984, Liebman [2] reported, for the first time, that DCP is highly sensitive to the diagnosis of hepatocellular carcinoma (HCC). With the improvement of DCP detection technology, its diagnostic and prognostic value for HCC has been confirmed by researchers [3, 4]. Although DCP is considered to be related to the prognosis of HCC, the relationship between DCP, DCP response, and prognosis of HCC patients treated with transarterial chemotherapy has become controversial in recent years.

Transcatheter arterial chemoembolization (TACE) and hepatic arterial infusion chemotherapy (HAIC) have become the main treatments for HCC tumors that cannot be excised [5]. These treatments can prolong the survival time of unresectable liver cancer. However, for chemotherapy-refractory patients, transarterial chemotherapy may delay but can also aggravate the patient's condition [6]. Therefore, it is important to predict the response to chemotherapy in patients at an early period of transarterial chemotherapy. In recent years, clinical trials have examined the relationship between decreased serum DCP levels after hepatic arterial chemotherapy and overall survival (OS) in patients with HCC, but the prognostic value remains controversial [7, 8]. In this study, we performed a meta-analysis to examine the existing evidence and determine whether low DCP values and DCP responses are related to better OS in patients with HCC undergoing transarterial chemotherapy.

## Materials and methods

We complied with the Preferred Reporting Items for Systematic Reviews and Meta-Analyses guidelines to conduct and report this meta-analysis after writing a study protocol [9].

### Literature search

A systematic review was performed using PubMed, the MEDLINE database, EMBASE, and the Cochrane Library independently by two authors (Ming Yang and Jinlong Liu). The databases were searched from January 1966 to May 2019 with the following terms: (((((“Des-gamma-carboxy prothrombin”) OR “des-γ-carboxy prothrombin”) OR DCP)) AND Hepatic carcinoma). Relevant full-text articles were subsequently reviewed and critiqued.

### Selection criteria

The inclusion criteria of this study were as follows: (i) Articles published in English between 1966 and 2019. (ii) Random or retrospective observational studies evaluating the prognostic value of DCP in hepatocellular carcinoma patients undergoing transarterial chemotherapy. (iii) The study provided sufficient data including hazard ratios (HRs) and 95% confidence intervals (CIs); if not, then data that can be used to calculate HRs was mandatory. (iv) HCC patients only received hepatic arterial infusion chemotherapy with or without transcatheter arterial embolization during the study period. The exclusion criteria were letters, reviews, case reports, conference abstracts, editorials, and expert opinion.

### Data extraction

Titles and abstracts were independently screened for relevance. Two independent investigators (Ming Yang, Jinlong Liu) extracted data from the included studies. Any divergences between the two reviewers about the selection of studies were discussed with the senior author (Xuejun Zhang) in order to include articles that best matched the protocol until consensus was reached. The following data were extracted: (i) Study characteristics: Author, year of publication, country, study design, sample size, therapeutic schedule, classification of HCC, OS, and progression-free survival (PFS). (ii) The OS and PFS were assessed as the primary measures of the treatment effect using the hazard ratio (HR) with 95% confidence interval (CI). The HR for the DCP baseline and DCP response was extracted. The DCP baseline is the value measured before treatment; the DCP response is defined as the patient's DCP value dropping by a certain amount after treatment.

### Level of evidence and quality assessment

The Newcastle–Ottawa Scale (NOS) [10] was used to assess study quality. The NOS consists of three parameters of quality: patient selection (0–4 points), study comparability (0–2 points), and outcome assessment (0–3 points). The maximum possible score is 9 points, representing the highest quality methodological study.

### Statistical analysis

We used STATA 15.0 software for data synthesis. The pooled HR and 95% CI were used to estimate the impact of DCP baseline and DCP response on survival. HR > 1 certified a worse survival for DCP baseline and DCP response. If HR > 1 represented a better prognosis in the included article, then inverted the HR was obtained by taking the reciprocal of the HR and associated CI [11] If a direct report of HR and 95% CI was not given, the estimated value was derived indirectly from Kaplan–Meier curves using the methods described by Tierney [11]. Heterogeneity among the studies was assessed by the Cochran's Q statistic and I^2^ tests. Either P < 0.10 or I^2^ statistic > 50% defined significant heterogeneity among the studies (0% to 50% represents low heterogeneity, 50% to 75% represents moderate heterogeneity, and 75% to 100% represents high heterogeneity). If significant heterogeneity existed, a random effects model was used; otherwise, a fixed effects model was implemented. To quantify the bias captured by the funnel plot and to formally assess reporting bias, we calculated the Egger’s regression intercept for outcomes reported by 6 studies.

## Results

### Article selection and patient demographics

**Fig 1:**
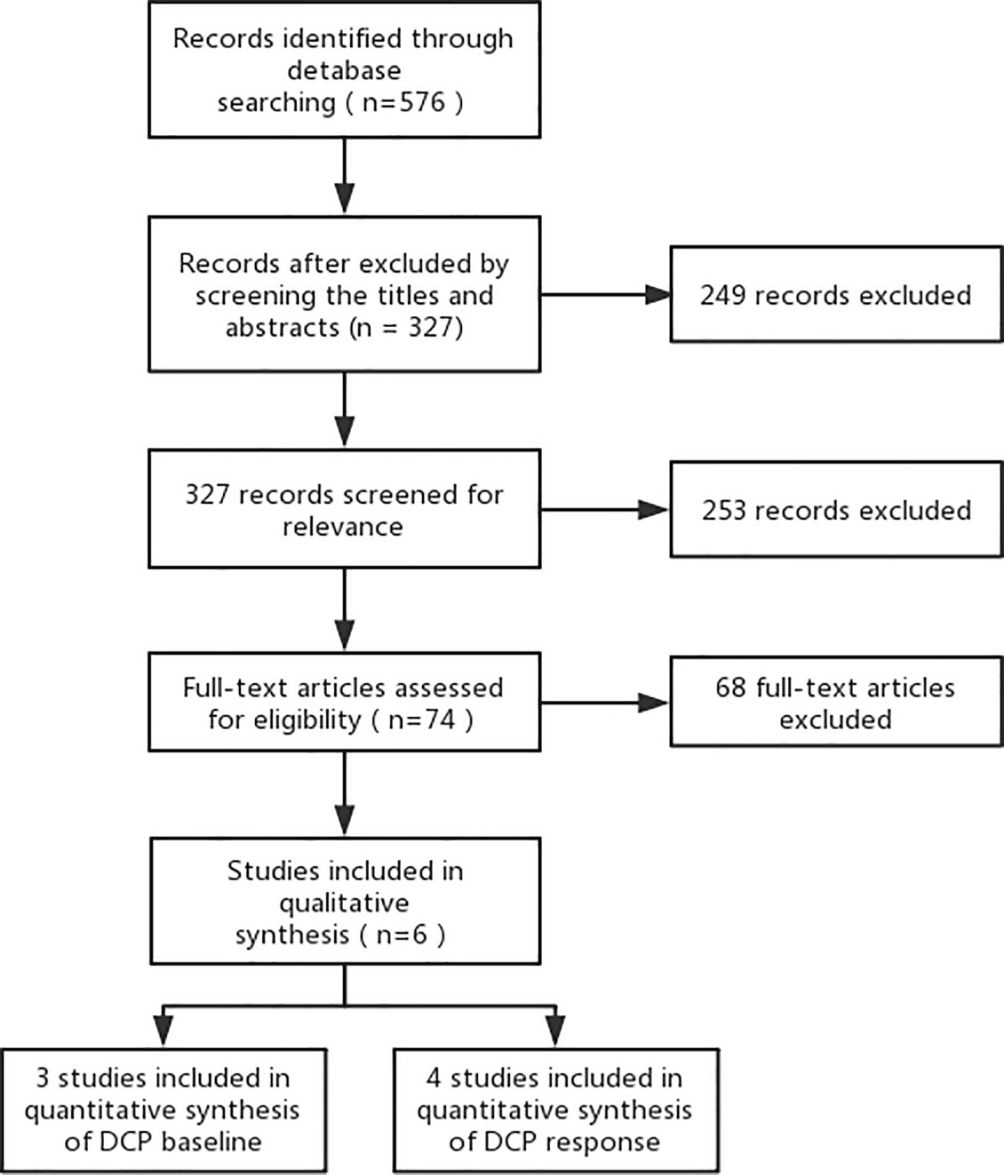
PRISMA flow chart represents the flow of information through the different phases of the systematic review.

A flow diagram of the systematic literature search is shown in Fig 1. Among the 576 articles in PubMed, Medline, EMBASE, and Cochrane Library Central that were retrieved, six articles were included in the quantitative synthesis [5, 7, 8, 12–14]. All of the six studies [5, 7, 8, 12–14] were retrospective in design. The included studies were conducted in Japan [5, 7, 13] and Korea [8, 12, 14] and were published between 2011 and 2018. The study sample sizes ranged from 90 to 327 patients. A total of 943 patients were treated with transarterial chemotherapy. A total of 712 (76%) patients from four studies [5, 8, 12, 13] underwent TACE and 231 (24%) patients from three studies [5, 7, 14] underwent HAIC. The baseline characteristics and Newcastle–Ottawa rating scale assessment of studies are provided in Table 1.

**Table 1:** Characteristics of included studies.

Author, year	No. of patients	Etiology (hepatitis/others)	Child-Pugh score (A/B)	Tumor size (cm)#	Pretreatment (+/-)	Treatment	DCP baseline (mAU/mL)#	DCP baseline cut-off value (mAU/mL)	DCP-positive response	Median overall survival (months)	Quality score
Issei Saeki, 2015 [7]	90	58/32	44/46	-	+	HAIC	1029.0(6.0–344740.0)	<1000	≥20%	10.6	7
Won-Hyeong Park, 2012 [12]	327	278/49	265 /62	4 (3–5.6)	-	TACE	114(42–350)	-	≥50%	20.8	7
Hiroki Nishikawa, 2014 [5]	226	106/39	100/45	5.4±3.0	-	TACE/TACI	9071.8±34906.2	<100	-	32.2/31.68	6
Myoung Ha Lee, 2011 [14]	60	52/8	60/0	11.0 (2.3–15.4)	-	HAIC	2000 (23–2000)	-	≥20%	9.0	6
Yong Kang Lee, 2013 [8]	115	98/17	108/7	47 (10–160)	-	TACE	231 (20–2000)	<200	≥50%	26.0	6
Haruki Kimura, 2017 [13]	125	92/33	99/26	4.0 (1.0–13.0)	-	TACE	188 (<30–1965)	<150	-	31.2	7

-: Not given.

DCP baseline cut-off value: A cut-off value set by the author to calculate DCP baseline HR.

DCP positive-response: DCP positive-response was defined as a reduction from baseline after transarterial chemotherapy.

#Data shown in the table are means (ranges) or means ± standard deviation.

Hepatitis: hepatitis B virus, hepatitis C virus, hepatitis.

HAIC: Hepatic arterial infusion chemotherapy

TACE: Transarterial chemoembolization

TACI: Transcatheter chemotherapy

Pretreatment history: Liver resection, radiofrequency ablation, liver transplantation.

Three articles [5, 7, 13] provided DCP baseline HR, and a total of four HR values were extracted. For one article [13], patients were classified into two groups according to different stages, and two HR values of the DCP baselines were obtained from multivariate analyses.

Four articles [7, 8, 12, 14] provided HR values of OS in DCP responders. DCP response was defined as a reduction of ≥ 20% from the baseline after transarterial chemotherapy in two articles [7, 14], and ≥ 50% in the other two articles [8, 12]. Three articles [8, 12, 14] provided HR values of PFS in DCP responders. The studies by Saeki [7] and YK Lee [8] set HR > 1 to represent a better prognosis, so we converted the HR values of the DCP baseline and DCP response. The articles from YK Lee [8] and MH Lee[14] didn’t provide HR values of the DCP positive-response directly, so the Kaplan–Meier curves in the article were read by Engauge Digitizer Version 4.1 (http://digitizer.sourceforge.net/), then the survival data determined from the Kaplan–Meier curves were entered in the spreadsheet appended to Tierney’s paper [11].

### Meta-analysis for the prognostic value (OS) of the DCP baseline

Three articles [5, 7, 13] estimated the association between the DCP baseline and OS of HCC patients receiving transarterial chemotherapy. A low DCP baseline was associated with good OS in three studies [5, 7, 13], and statistical significance was observed in two studies [5, 13]. It is noteworthy that no statistical significance was observed in two studies [7, 13], since one study [13] divided patients into two groups according to different stages. Kimura [13] showed that DCP < 150(mAU/mL) detected in the early stage was not an independent factor predicting OS (P = 0.322).

**Fig 2:**
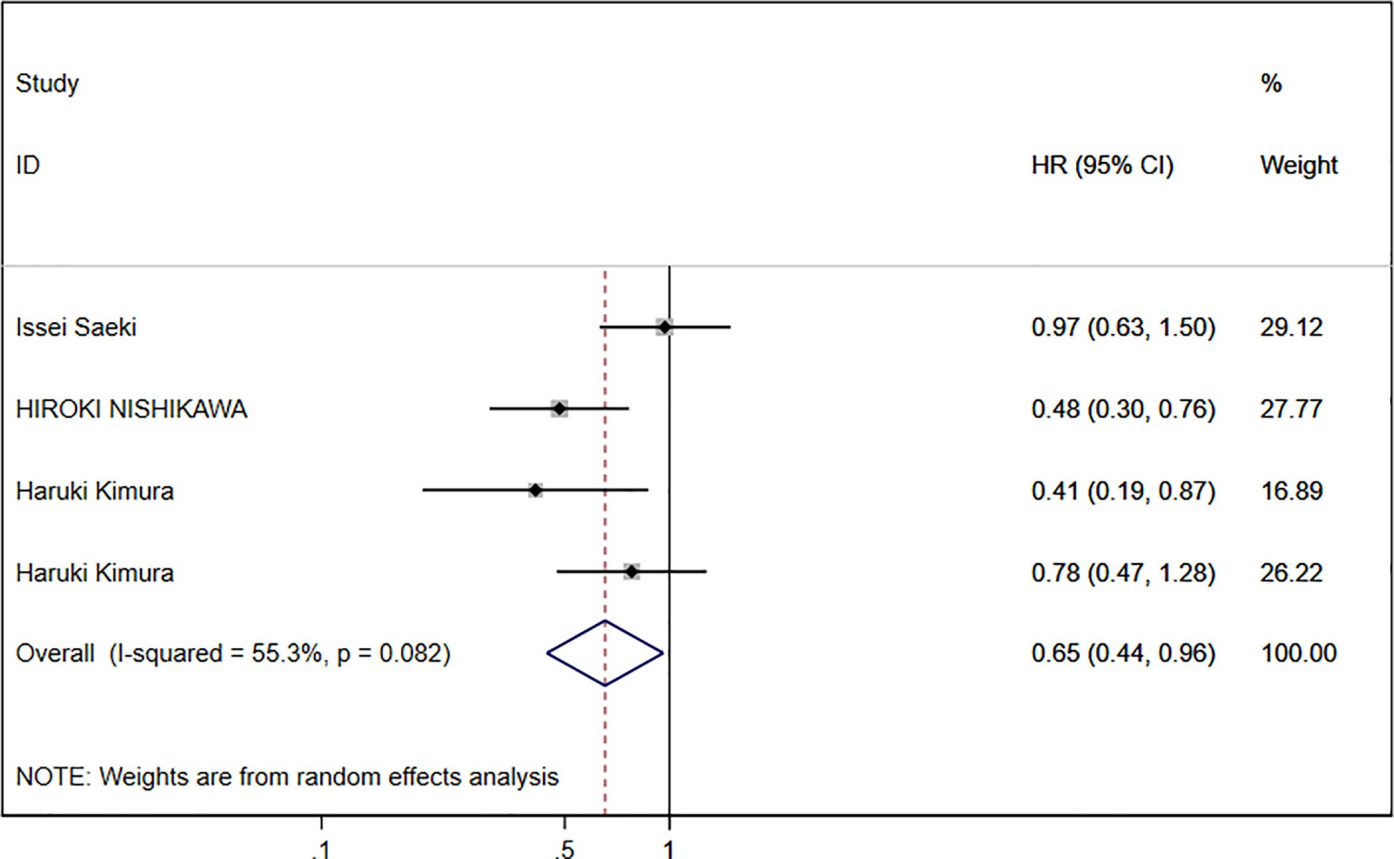
Forest plot of three studies with HR of DCP baseline for OS.

Combined data from the three [5, 7, 13] studies indicated that a low DCP baseline was significantly correlated with OS. A random effects model was used to pool all the included studies (Fig 2), and that demonstrated a low DCP baseline was associated with a favorable OS with an HR value of 0.653 (95% CI: 0.444–0.960) with a moderate heterogeneity (I^2^ = 55.3%, P = 0.08).

**Fig 3:**
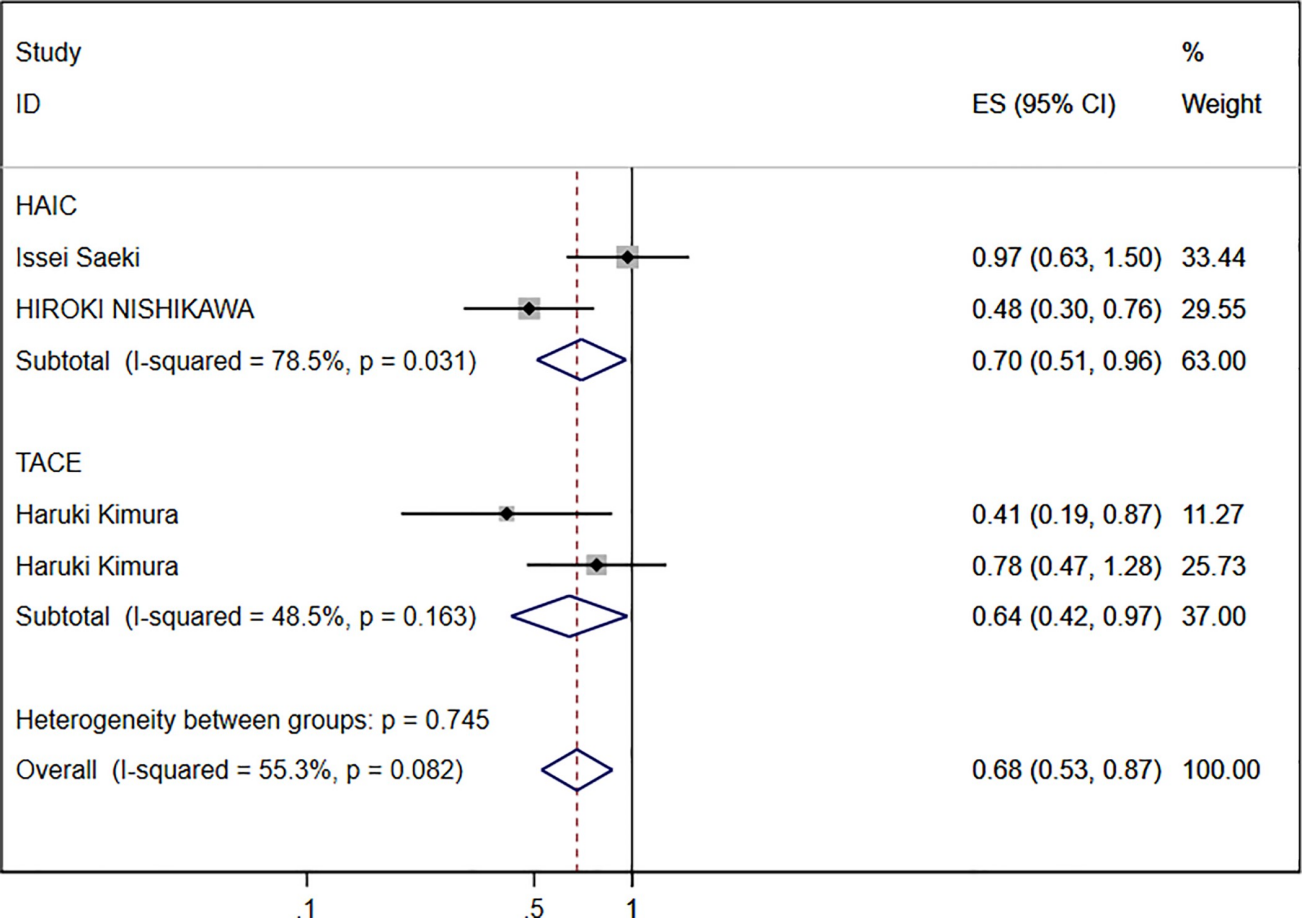
Subgroup analyses of three studies with HR of DCP baseline for OS.

According to the different treatments (TACE [13] and HAIC [5, 7]) used in the included literature, subgroup analysis was performed to observe the prognostic value of the DCP baseline. Patients with a low DCP baseline showed favorable results (HR 0.70, 95% CI 0.51–0.96) in the HAIC group. Similar results were observed in the TACE group (HR 0.64, 95% CI 0.42–0.97). Both pooled HRs indicated that patients with HCC had a favorable prognosis with a low DCP baseline after receiving transarterial chemotherapy (Fig 3).

### Meta-analysis for the prognostic value (OS) of the DCP response

The DCP response refers to a decrease of serum DCP values from the baseline of more than 20% [7, 14] or 50% [8, 12] in patients with HCC after arterial chemotherapy. The detection time was 2 weeks [7], 1 month [12], 2 months [14], or 2–3 courses [8] after HAIC or TACE.

**Fig 4:**
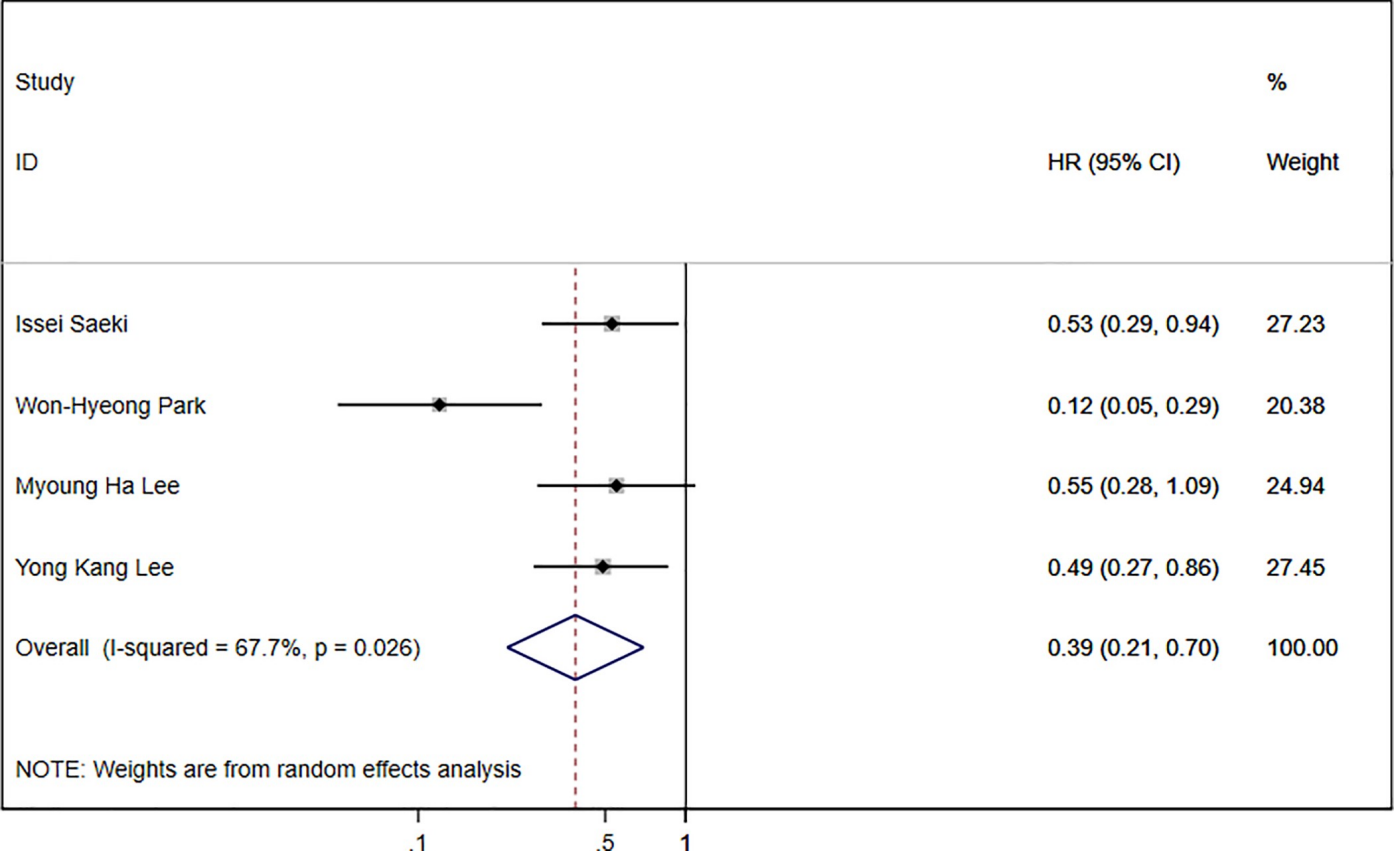
Forest plot of four studies with HR of DCP response for OS.

Four studies evaluated the correlation of the DCP response with OS in 592 HCC patients [7, 8, 12, 14]. The maximum follow-up period in these four articles was 26 months. The DCP response was correlated with increased OS in all four studies, but no statistical significance was observed in one study [14]. The combined HR for the four studies was 0.387 (95% CI: 0.215–0.697) with moderate heterogeneity (I^2^ = 67.7%, P = 0.03). The pooled result indicated that the DCP response was associated with increased OS in HCC (Fig 4).

Because of the different chemotherapeutics, DCP value detection time, lengths of follow-up, and the inconsistency of clinicopathological features, heterogeneity was still detected while analyzing the relationship between the DCP baseline, DCP response, and OS in HCC patients after transarterial chemotherapy. In this case, a random effect model was selected.

**Fig 5:**
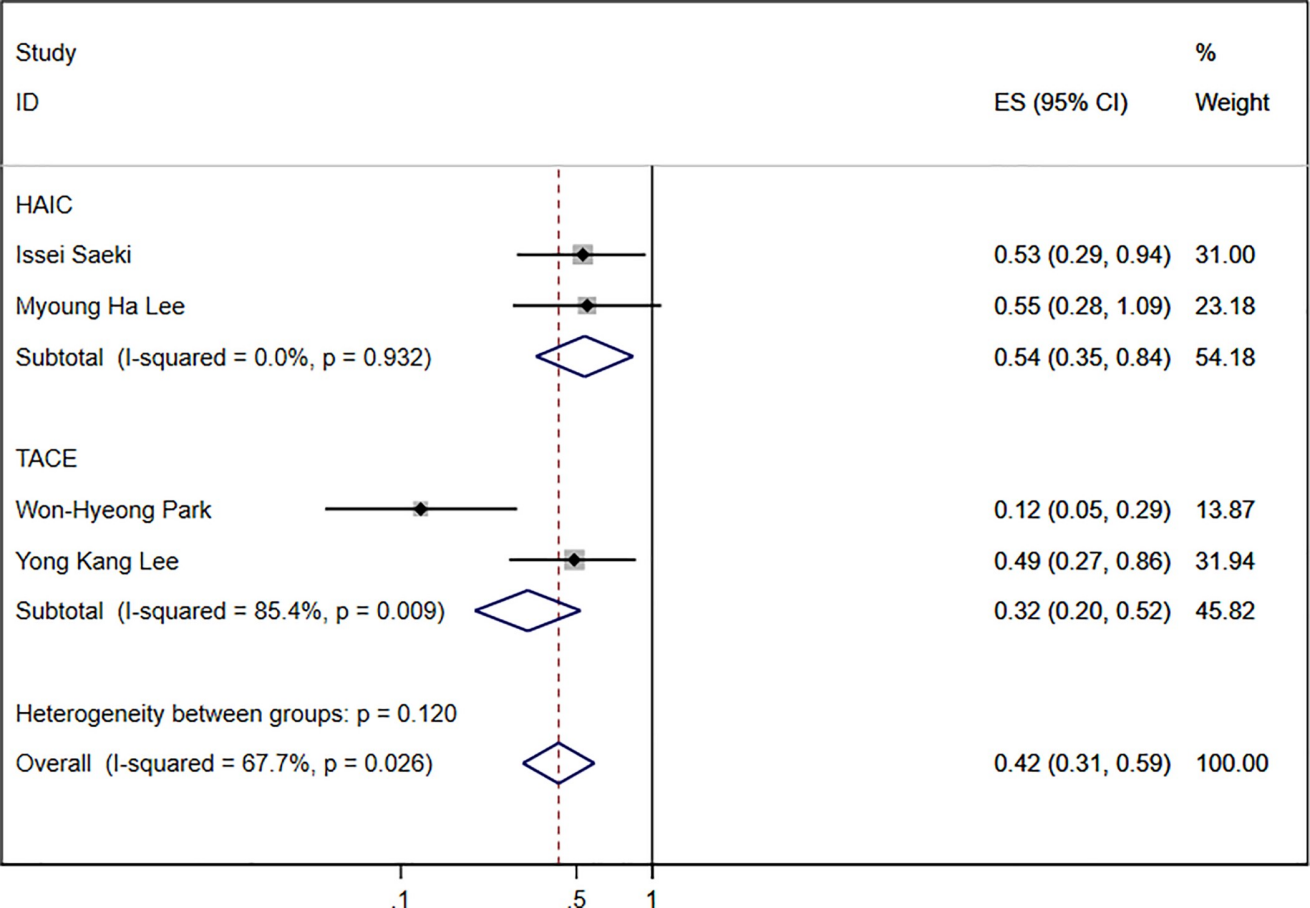
Subgroup analyses of four studies with HR of DCP response for OS.

Subgroup analysis was performed based on the different treatments, and HR values of the TACE group [8, 12] and HAIC group [7, 14] were respectively pooled. The results did not change in the subgroup analysis of treatments. The DCP response was of benefit to the prognosis of HCC patients receiving transarterial chemotherapy (TACE group: HR 0.32; 95% CI:0.20–0.52; HAIC group: HR 0.54; 95% CI:0.35–0.84; Fig 5).

### Meta-analysis for the prognostic value (PFS) of the DCP response

**Fig 6:**
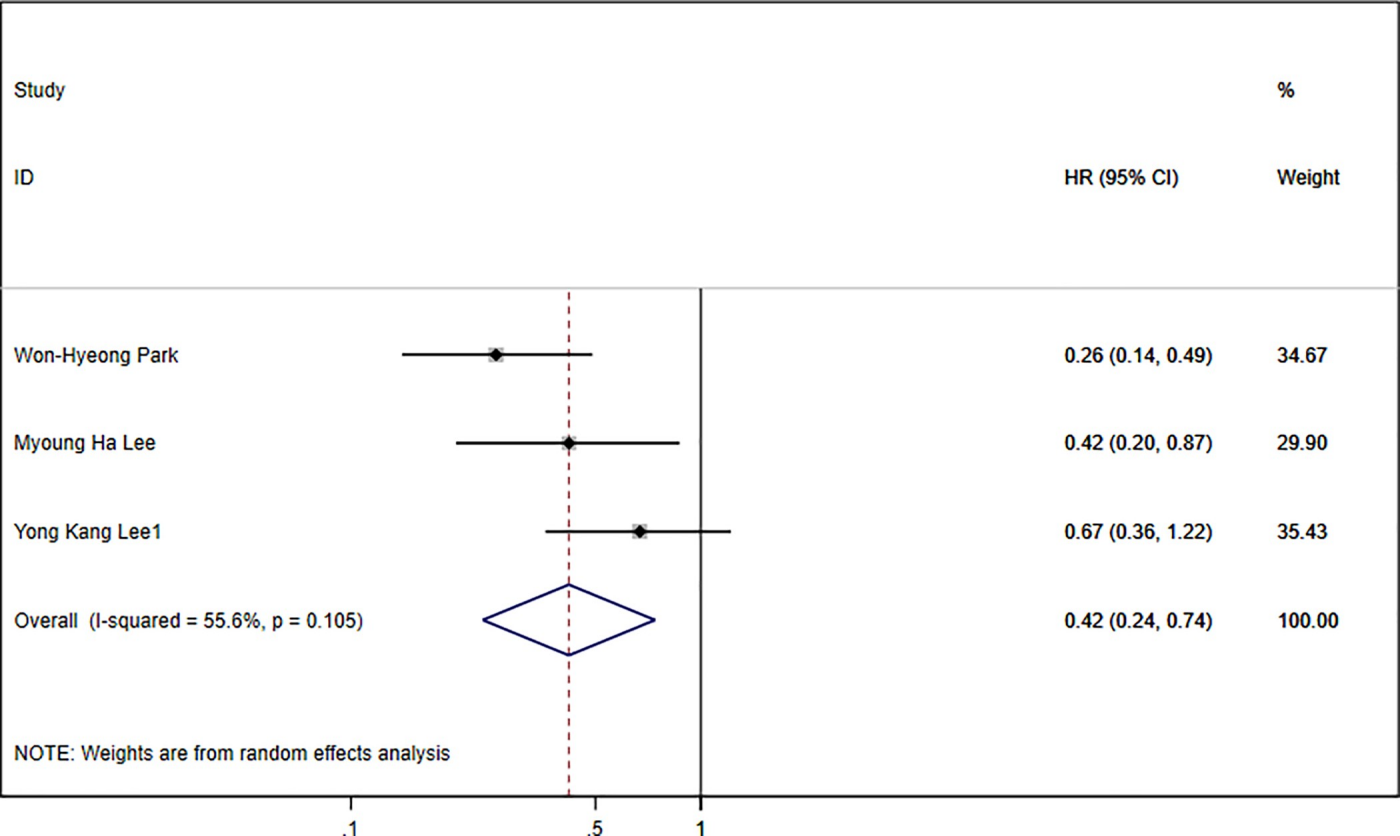
Forest plot of three studies with HR of DCP response for PFS.

Three articles [8, 12, 14] were included to investigate the relationship between the DCP response and PFS in patients with HCC receiving transarterial chemotherapy. Considering the significant heterogeneity (I^2^ = 55.6%; P = 0.105) between studies, we employed a random effect model to pool the HR. Pooled data showed that patients with a DCP response exhibited extended PFS with a combined HR of 0.42 (95% CI:0.23–0.74; Fig 6).

## Assessment of heterogeneity

### Risk of bias across studies

**Fig 7:**
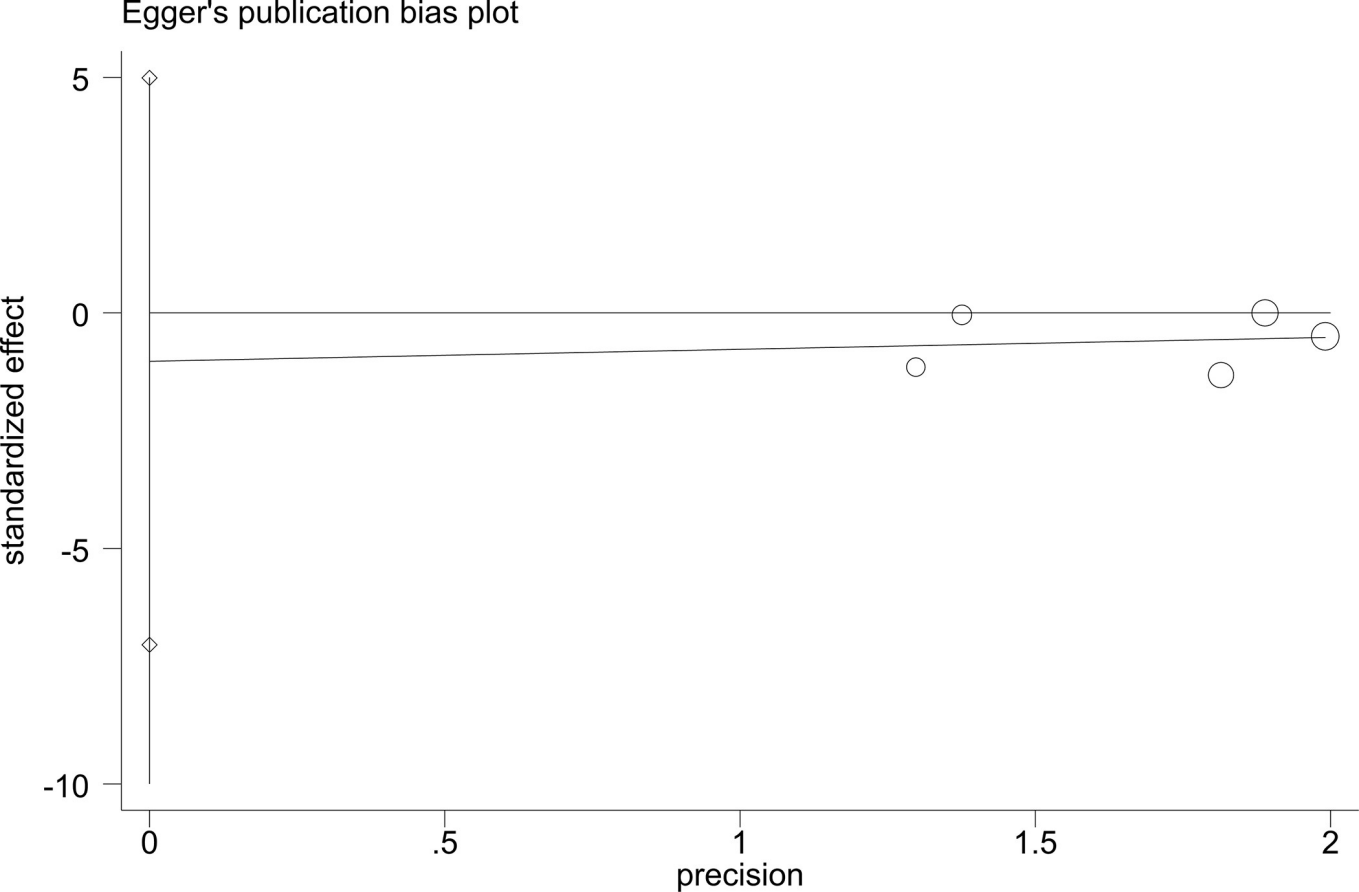
Egger’s test for DCP baseline.

**Fig 8:**
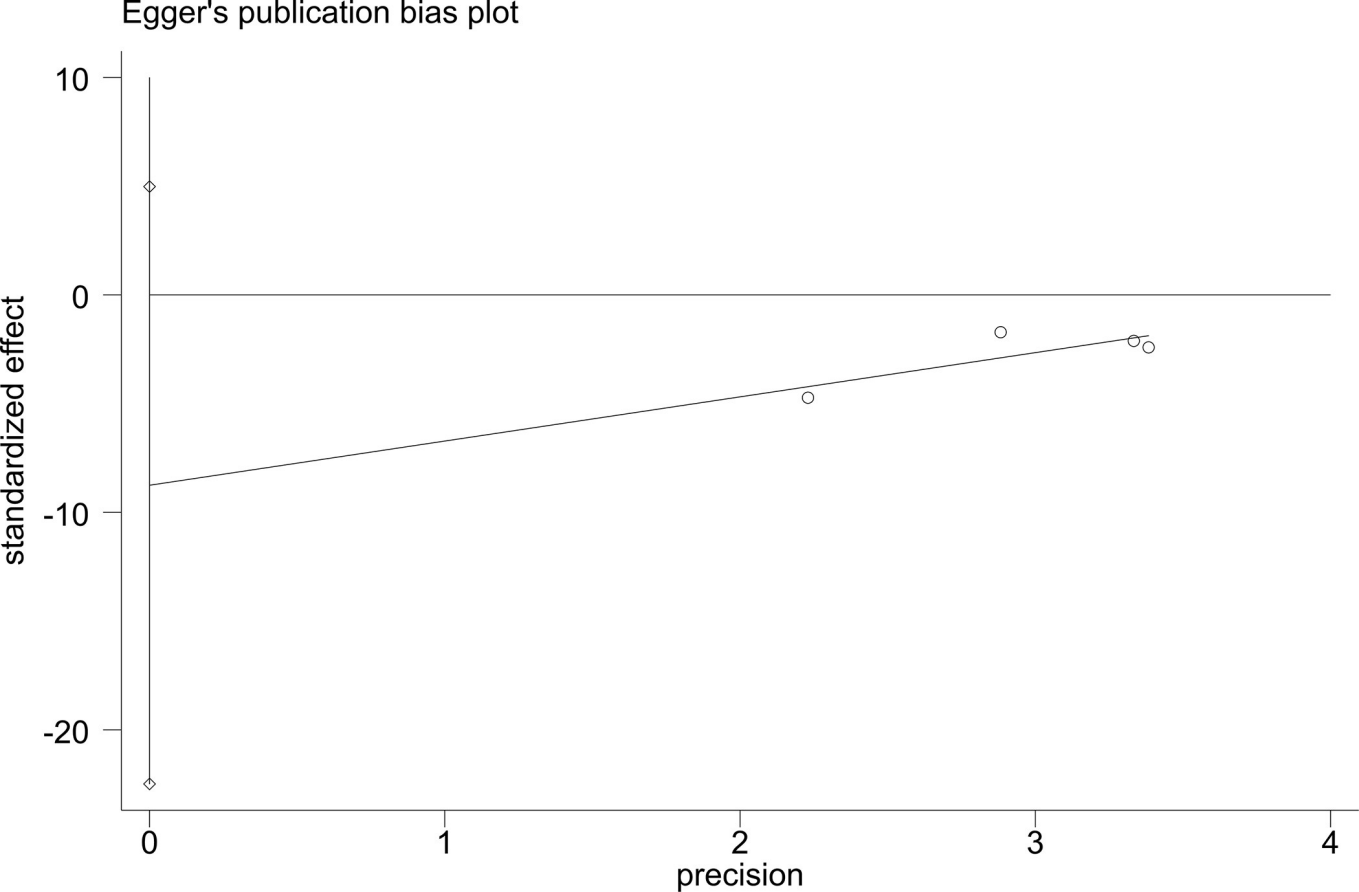
Egger’s test for DCP response.

To evaluate publication bias, we performed Egger’s test. The Egger test gave a P value of 0.624 for the DCP baseline and a P value of 0.111 for the DCP response, indicating no evidence of publication bias (Figs 7 and 8).

## Discussion

Our meta-analysis of six retrospective studies with 943 patients clearly showed that a low DCP baseline and DCP response were significant indicators of favorable OS and PFS for hepatocellular carcinoma patients with transarterial chemotherapy. Regardless of the varied baselines of DCP, if the DCP value decreases significantly, it may indicate that transarterial chemotherapy is effective and may control the progression of HCC to prolong the survival time of patients.

DCP is an immature prothrombin. In liver cancer cells, a lack of vitamin K or obstacles to the utilization of vitamin K result in a decrease in the activity of vitamin K-dependent enzymes, thereby producing DCP [15]. High serum DCP is positively correlated with tumor burden in patients with HCC [16]. The higher the serum DCP value in liver cancer patients, the worse the degree of differentiation of liver cancer [17], and the incidence of portal vein invasion [18] and intrahepatic metastasis [19] also increases. Furthermore, previous studies have found that high DCP values are a risk factor for recurrence after hepatectomy in patients with hepatitis B-related liver cancer [20].

Transarterial chemotherapy is an effective method for the treatment of advanced HCC [21]. Transarterial chemotherapy refers to the injection of embolic agents and chemotherapeutics into the nourishing arteries of tumors, causing tumor tissue ischemia and necrosis. At the same time, chemotherapeutics act directly on the lesion and kill the cancer cells.

Some clinical features of patients with HCC have been found to have important predictive value for their prognosis when treated with transarterial chemotherapy, including high AFP values, high Child–Pugh scores [22], and presence of portal vein invasion [23]. However, the role of DCP in predicting the prognosis of patients with liver cancer undergoing transarterial chemotherapy remains controversial.

Our meta-analysis was performed to determine the relationship between DCP values and the prognosis of patients with HCC who underwent transarterial chemotherapy. Our findings suggest that HCC patients with low DCP survived longer than those with high DCP. Thus, we conclude that high DCP is a risk factor for poor prognosis in HCC patients after receiving transarterial chemotherapy.

For advanced HCC patients, the course of transarterial chemotherapy is usually repeated two to three or more times. Furthermore, a portion of patients who are refractory to chemotherapy will have poor prognosis [7]. Contrast CT is usually used to evaluate the therapeutic efficacy of transarterial chemotherapy [24, 25]. However, inhomogeneous iodized oil deposition within tumors and masses with liquefied necrotic areas of low density after chemoembolization can interfere with the measurement accuracy of the longest diameters of enhanced target lesions [26, 27]. Moreover, during the early stage of treatment, the reduction of tumor burden and pathologic remission do not always result in significant radiological changes[28].

In this study, the meta-analysis for DCP response indicated that it is an effective prognostic factor for better survival in patients who have received transarterial chemotherapy, including TACE and HAIC. The survival time in the DCP response group was longer than in the non-DCP response group. This finding suggests that changes in serum DCP in patients before and after transarterial chemotherapy can reflect the therapeutic effect and may be useful in clinical decision making and prognosis. A previous study showed that the survival time of chemotherapy-refractory patients is significantly shorter than in patients who respond to chemotherapy; if chemotherapy-refractory patients are immediately given sorafenib to replace current chemotherapeutics, patient prognosis clearly improves [29].

The first time that the DCP value was monitored after transarterial chemotherapy in the included studies was always at an early course of treatment. Our meta-analysis for DCP response confirmed the recommendation to use it as an auxiliary method to assess drug sensitivity in the clinic. This finding may have pragmatic implications. Using the DCP response may reflect the chemotherapeutic effect at an early stage and provide physicians with important information that would facilitate decisions to replace existing chemotherapeutics or therapeutic regimens.

However, there are certain limitations in this meta-analysis, which should be acknowledged. First, only English-language literature reports were included in this analysis, so there may have been some bias due to the language criteria. Second, if HRs and their 95% CIs were not directly given in an article, they were calculated using the Kaplan–Meier survival curves according to the methods mentioned above. In this process, distorted data could have been accepted, resulting in reduced credibility of the results. Furthermore, the included studies used different staging systems for HCC, which can impact the results. Finally, all patients in the selected studies were from Asia (Japan, Korea, and Thailand) where HCC patients are mainly HBV infected, and these studies are retrospective in nature. Thus, the present results should be updated with more studies from Western countries in the future.

## Conclusion

Based on our meta-analysis, low DCP and the DCP response were associated with good prognosis in HCC patients who underwent transarterial chemotherapy. Changes in serum DCP in patients before and after transarterial chemotherapy can reflect the therapeutic effect and may be useful in clinical decision making.

## Supporting information

S1 PRISMA Checklist (DOC)
